# *Paracoccus broussonetiae* subsp. *drimophilus* subsp. nov., a Novel Subspecies Salt-Tolerant Endophytic Bacterium from Maize Root in Hunan

**DOI:** 10.3390/life15030354

**Published:** 2025-02-24

**Authors:** Xue Li, Chi Zhou, Ming Li, Qingzhuang Zhang, Lei Su, Xin Li

**Affiliations:** 1 Hunan Institute of Microbiology, Changsha 410009, China; swine91@163.com (C.Z.); qing90202412@163.com (Q.Z.); 2 National Human Diseases Animal Model Resource Center, Institute of Laboratory Animal Sciences, Chinese Academy of Medical Sciences (CAMS) & Peking Union Medical College (PUMC), Beijing 100021, China; lixue@cnilas.org; 3 Hunan Engineering Research Center for Endophytic Microbial Resources Mining and Utilization, Changsha 410125, China; 4 Institute of Animal Sciences of Chinese Academy of Agricultural Sciences, Beijing 100193, China; liming01@caas.cn

**Keywords:** endophytic bacterium, polyphasic taxonomy, salt-tolerant bacterium

## Abstract

In an investigation exploring endophytic microbiota from agricultural crops, an aerobic, non-motile, Gram-negative, coccobacillus-shaped bacterial isolate, designated as strain NGMCC 1.201697^T^, was isolated from maize roots in Hunan Province, China. Phylogenetic analysis based on 16S rRNA gene sequences revealed that strain NGMCC 1.201697^T^ belonged to the genus *Paracoccus*, showing the highest sequence similarity to *Paracoccus broussonetiae* CPCC 101403^T^ (99.86%). The average nucleotide identity (ANI) and digital DNA–DNA hybridization (dDDH) were 98.57% and 87.90% between the novel isolate and its closest phylogenetic relative. However, phenotypic characterization further differentiated the isolate from *P. broussonetiae* CPCC 101403^T^. The isolate showed enhanced environmental tolerance adaptability (growth in 0–8% NaCl and 4–37 °C), unique enzymatic activities (esterase C4, β-glucosidase, L-proline arylamidase, and β-galactosidase), and expanded metabolic capabilities (D-mannitol, D-cellobiose, saccharose, and so on). The major polar lipids consisted of diphosphatidylglycerol (DPG), phosphatidylethanolamine (PE), phosphatidylcholine (PC), phosphatidylglycerol (PG), two unidentified glycolipids (GLs) and four unidentified phospholipids (PLs). The predominant respiratory quinone was ubiquinone-10, and the major fatty acid was summed feature 8 (C18:1 ω7c, 69.42%). The DNA G + C content was 64.49 mol%. Based on results of these analyses, strain NGMCC 1.201697^T^ represents a novel subspecies of *Paracoccus broussonetiae*, for which the name *Paracoccus broussonetiae* subsp. drimophilus subsp. nov. is proposed. The type-strain is NGMCC 1.201697^T^ (=CGMCC 1.61958^T^ =JCM 37104^T^).

## 1. Introduction

The genus *Paracoccus*, belonging to the family *Rhodobacteraceae*, order *Rhodobacterales*, within the *Proteobacteria* phylum, was first reported by Davis et al. (1969) [1] and subsequently emended by Ludwig et al. [2] and Liu et al. (2008) [3]. *Paracoccus denitrificans* has been recognized as the type species. Members of this genus are characterized as Gram-negative, coccoid-shaped bacteria (0.4–0.9 μm in diameter and up to 2 μm in length) with variable motility. Ubiquinone-10 is the dominant respiratory quinone, summed feature 8 is the major fatty acid, and a high DNA G + C content (63–71 mol%) is one of the chemotaxonomic features. In December 2024, the LPSN database (https://lpsn.dsmz.de/genus/paracoccus) [4] acknowledged 112 validly published species in this genus. *Paracoccus* spp. demonstrate remarkable metabolic versatility and ecological adaptability, having been isolated from diverse environments worldwide, such as soil [5,6,7,8,9], air [10], various host organisms [11,12], activated sludge from water or marine sediments [13,14] and bioreactors [15,16,17]. Their widespread distribution reflects their exceptional adaptability to different environmental conditions. Their ability to grow both heterotrophically and autotrophically, utilizing various organic and inorganic compounds as carbon and energy sources, enables them to adapt to various environmental niches and participate in global carbon and nitrogen cycles. This metabolic diversity has attracted considerable attention for their potential applications in biotechnology and environmental remediation, and the continuing discovery of new species suggests that the full diversity and potential of this genus are yet to be fully understood.

Plant-associated microorganisms, particularly those adapted to extreme conditions, play crucial roles in regulating plant growth, crop productivity, and stress tolerance [18]. These microorganisms enhance plant stress tolerance through various mechanisms, including the production of plant growth-promoting compounds, osmolytes, and stress-responsive enzymes [19]. Of particular interest are salt-tolerant bacteria. Salt-tolerant microorganisms have shown promising results in promoting plant growth under saline conditions through mechanisms such as the production of ACC deaminase, IAA, and exopolysaccharides [20]. Psychotolerant microorganisms, on the other hand, possess cold-active enzymes and unique membrane modifications that enable their survival at low temperatures [21]. Recent studies have demonstrated their potential in enhancing cold tolerance in crops through various mechanisms including cold shock proteins (CSPs) and cold acclimation proteins (CAPs) [22]. In our study, strain NGMCC 1.201697^T^ exhibits dual characteristics of halophilicity and low temperature growth. The identification of novel strains stress tolerance capabilities is, therefore, of significant agricultural importance.

In this study, we isolated strain NGMCC 1.201697^T^ from maize root in Hunan Province. Initial characterization suggested this strain belongs to the genus *Paracoccus* and possesses notable stress tolerance characteristics. The present study aims to determine the taxonomic position of strain NGMCC 1.201697^T^ through a comprehensive polyphasic approach, including phenotypic, chemotaxonomic, phylogenetic, and genomic analysis. As a result, strain NGMCC 1.201697^T^ was identified as a novel subspecies of the genus *Paracoccus*, for which the name *Paracoccus broussonetiae* subsp. *drimophilus* subsp. nov. was proposed.

## 2. Materials and Methods

### 2.1. Isolation, Cultivation, and Preservation

Strain NGMCC 1.201697^T^ was isolated from maize roots collected from Hunan Province (28°08′23″ N, 112°49′04″ E), China, on 5 August 2023. Fresh root samples were washed thoroughly with sterile saline (0.9%, *w*/*v*, NaCl). After surface moisture was removed, samples (2–3 g) underwent surface sterilization that included immersion in 5% (*v*/*v*) sodium hypochlorite solution (1 min) and 75% (*v*/*v*) ethanol (1 min), followed by triple-rinsing with filter-sterilized distilled water. Sterilized samples were dried on sterile filter paper and homogenized in 10 mL of aseptic distilled water with an autoclaved mortar and pestle. The homogenate was left to stand for 2–3 min before the supernatant was serially diluted (10^−1^ to 10^−7^). Aliquots (100 μL) of 10^−5^ to 10^−7^ dilution were spread on PDA plates (Hopebio, China) and incubated at 28 °C for at least 7 days. Isolated colonies were purified through repeated streaking on fresh PDA plates. Cultures were stored long-term in 20% (*v*/*v*) glycerol at −80 °C.

### 2.2. Morphological, Physiological, and Biochemical Characterization

Cell morphology was observed using optical microscopy (BX53; Olympus, Tokyo, Japan) and SEM (scanning electron microscopy) (H-7650; Hitach, Tokyo, Japan) with LB agar-grown cells incubated for 48 h at 30 °C. Gram staining involved a commercial Gram-stain kit (G1060; solarbio, Beijing, China), applied in accordance with the manufacturer’s instructions. Growth under anaerobic conditions was tested using an anaerobic chamber (Forma Scientific, Marietta, OH, USA) with N_2_/CO_2_/H_2_ (90:5:5, *v*/*v*) atmosphere. The temperature range for growth and the optimum temperature was determined on LB agar at 4, 10, 20, 25, 30, 37, 45 and 50 °C. Tolerance to pH (3.0–11.0, increments of 1.0 pH unit, adjusted with HCl or NaOH) and NaCl (0–15%, *w*/*v*, increments of 1.0%) were evaluated in both LB broth and agar. Catalase activity was assessed by observing bubble formation in 3% (*v*/*v*) hydrogen peroxide (H_2_O_2_) solution. Oxidase activity and other biochemical characteristics were determined using API 20NE, API ZYM strips, and VITEK^®^ 2 GN cards (bioMérieux, Marcy-l’Étoile, France), according to the manufacturer’s instructions. Reference strains were tested in parallel under identical conditions.

For chemotaxonomic characterization, cells were cultivated on LB agar plates at 30 °C for 48 h during their exponential phase. Following the MIDI protocol (Microbial Identification System, version 6.0) described by Sasser (1990) [23], fatty acid methyl esters (FAMEs) were extracted, analyzed and identified using the TSBA 6 database. Using the method by Minnikin et al. [24], polar lipids were extracted and resolved via two-dimensional thin-layer chromatography (TLC). The solvents chloroform–methanol–water (65:25:4, *v*/*v*) and chloroform–methanol–acetic acid–water (80:12:15:4, *v*/*v*) were applied sequentially for the first and second dimensions. Polar lipids were detected by spraying with specific reagents: molybdophosphoric acid for total lipids, α-naphthol for glycolipids, ninhydrin for aminolipids, molybdenum blue for phospholipids, and Dragendorff’s reagents for choline-containing lipids (Sigma-Aldrich, St. Louis, MO, USA). Quinones fractions were obtained from the lyophilized cells through chloroform–methanol (2:1, *v*/*v*) extraction and purified using preparative TLC developed in hexane–diethyl ether (34:6, *v*/*v*). Quantification of quinones was achieved through reversed-phase HPLC, carried out on an Elite HPLC system. Separation on an octadecylsilane column (ODS 5 mm, 4.6 × 150 mm, I, d) was coupled with detection at wavelengths of 275 nm and 270 nm for ubiquinones and menaquinones, respectively.

### 2.3. Phylogenetic Analysis

Genomic DNA was extracted from strain NGMCC 1.201967^T^ using a bacterial genomic DNA kit (TIANGEN, Beijing, China), according to the manufacturer’s instructions. PCR amplification of the 16S rRNA gene was conducted with PrimeSTAR^®^ HS DNA polymerase (Takara, Dalian, China), with universal primers 27F/1492R [25]. PCR products were subjected to sequencing at BGI Genomics Co., Ltd. (Shenzhen, China), and the resulting 16S rRNA sequence was aligned against the NCBI RefSeq database through BLASTn analysis [26]. Multiple sequence alignment was performed using Clustal_X version 2.0 to ensure the proper alignment of homologous sites [27]. Phylogenetic topology reconstruction employed three independent approaches: neighbor-joining (NJ) [28], maximum-likelihood (ML) [29], and maximum-parsimony (MP) in MEGA_X version 11.0 [30]. Evolutionary distances were computed using the Kimura two-parameter model [31]. Nodal confidence was assessed by 1000 bootstrap re-sampling for all methods.

### 2.4. Genome Sequencing and Analysis

Genome sequencing of strain NGMCC 1.201967^T^ was sequenced at BGI (Beijing Genomics Institute, Shenzhen, China) on the DNBSEQ platform. Quality control of raw sequencing data were performed using SOAPnuke version 1.5.6 [32]. Following data filtering, assembly procedures of clean reads were executed through SOAP denovo version 2.04 [33], with subsequent assembly quality evaluated by QUAST version 5.0.2 [34]. Gene prediction utilized Glimmer version 3.02 [35]. Non-coding RNA components were characterized through tRNAscan-SE for transfer RNA [36], RNAmmer for ribosomal RNA, and the Rfam database queries for small RNA. To elucidate the functional capabilities of the genome, comprehensive functional annotation was conducted against multiple databases: KEGG (Kyoto Encyclopedia of Genes and Genomes), COG (Clusters of Orthologous Groups), NR (Non-Redundant Protein Database databases), Swiss-Prot, and GO (Gene Ontology).

### 2.5. Phylogenomic Analysis

Phylogenomic analysis was conducted through marker gene identification using GTDB-Tk v2.3.0 [37]. The protocol employed specialized hidden Markov models (HMM) profiles to extract single-copy proteins (120 bacterial/122 archaeal markers) according to the GTDB reference pipeline (Release R214) [38]. Sequence processing incorporated multiple steps: marker proteins underwent positional filtering (minimum 50% occupancy requirement), followed by PyNAST-based alignment optimization. The resulting concatenated alignment yielded a comprehensive protein matrix spanning 10,432 amino acid positions. The maximum-likelihood phylogenetic tree was reconstructed using IQ-TREE v2.0 with the LG+F+G4 substitution model. Node support was assessed using 1000 ultrafast bootstrap replicates and SH-aLRT branch tests. Genomic similarities were evaluated using average nucleotide identity (ANI) and digital DNA–DNA hybridization (dDDH). ANI was calculated using the OrthoANI algorithm implemented in EzBioCloud [39], and dDDH was estimated using the GGDC 3.0 algorithm on the Type-strain Genome Server (TYGS) [40].

## 3. Results and Discussion

### 3.1. Physiological and Chemotaxonomic Characteristics

**Figure 1:**
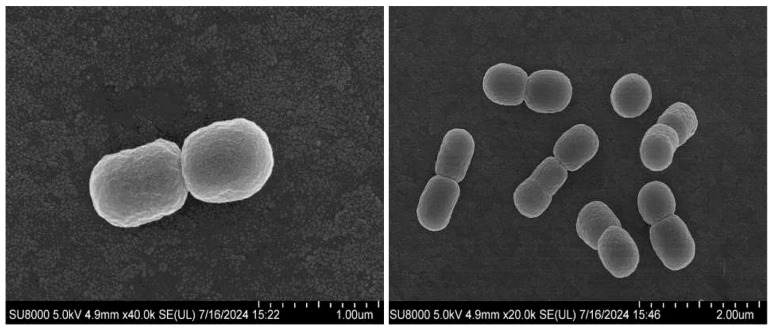
Transmission electron micrograph (TEM) image of strain NGMCC 1.201697^T^ during the exponential stage of growth. Bar, 1 μm and 2 μm.

Following 48 h incubation on LB agar at 30 °C, colonies of strain NGMCC 1.201697^T^ exhibited as yellowish-white, circular, glistening, and convex with intact edges, measuring 1.0–2.0 mm in diameter. Microscopic and SEM inspection showed that non-motile, Gram-negative, coccobacillus bacteria in pairs, measuring 0.60–0.85 μm (length) × 0.55–0.60 μm (width) under aerobic conditions (Figure 1). In contrast to the semitranslucent colonies observed in *P. broussonetiae* CPCC 101403^T^, strain NGMCC 1.201697^T^ consistently formed yellowish-white colonies. Table 1 provides a comparative description of the physiological and chemotaxonomic properties distinguishing strain NGMCC 1.201697^T^ from four *Paracoccus* type-strains: *P. broussonetiae* CPCC 101403^T^ [41], *P. litorisediminis* NBRC 112902^T^ [42], *P. yeei* CCUG 46822^T^ [43,44] and *P. denitrificans* ATCC 19367^T^ [2,44,45], selected on the basis of phylogenetic analysis and phylogenomic tree topology. Strain NGMCC 1.201697^T^ demonstrated an enhanced capacity to withstand variable environmental conditions compared to four *Paracoccus* type-strains, particularly regarding salinity, temperature, and pH adaptability. On salinity, NGMCC 1.201697^T^ tolerated up to 8% NaCl (optimum 0–2%), markedly exceeding the performance of *P. broussonetiae* CPCC 101403^T^, which was limited to 5% NaCl (optimum 0–1%). This elevated saline tolerance suggests that NGMCC 1.201697^T^ may have evolved a higher tolerance for saline concentrations based on the core functionality of the same species, thereby gaining a competitive survival advantage in more extreme environments. On temperature, while all tested strains exhibited an optimal growth temperature of 30 °C, NGMCC 1.201697^T^ uniquely displayed cryotolerance by growing at 4 °C. This unique cold adaptation may result from specific gene regulatory mechanisms or changes in membrane composition. On pH adaptation, NGMCC 1.201697^T^ also maintained growth over a wider pH range (4.0–10.0, optimum 7.0) compared to *P. broussonetiae* CPCC 101403^T^ (7.0–9.0). This expanded pH tolerance underscores its flexibility under acidic and alkaline conditions. Members of the *Paracoccus* genus are widely recognized for distinct physiological features like its halophilic and halotolerant nature and broad pH range. Notably, NaCl tolerance varies within the genus, with certain species, such as *P. halophilus* [3] and *P. saliphilus* [46], identified as strictly halophilic. In contrast to other primarily species, strain NGMCC 1.201697^T^ exhibits remarkable low-temperature growth. All tested strains were positive for oxidase, L-malate, catalase, naphthol-AS-BI-phosphohydrolase, and assimilation of D-glucose and D-mannose, while being uniformly negative for β-glucuronidase, lipase, α-fucosidase, gelatin hydrolysis, α-mannosidase, β-glucosidase, chymotrypsin, α-galactosidase, lipoidase (C14) and β-galactosidase. Distinctively, strain NGMCC 1.201697^T^ uniquely exhibited additional enzymatic activities, esterase (C4), β-glucosidase, L-proline arylamidase, and β-galactosidase, which were absent in *P. broussonetiae* CPCC 101403^T^, serving as key differentiators at the subspecies level. Furthermore, NGMCC 1.201697^T^ metabolized a broader range of substrates, including D-mannitol, D-maltose (weakly positive), D-cellobiose, D-sorbitol, saccharose/sucrose, and D-trehalose compared to the more limited substrate utilization observed in *P. broussonetiae* CPCC 101403^T^. These distinctions further underscore significant ecological and functional divergence between the two strains.

### 3.2. Phylogenetic Analyses Based on 16S rRNA

**Figure 2:**
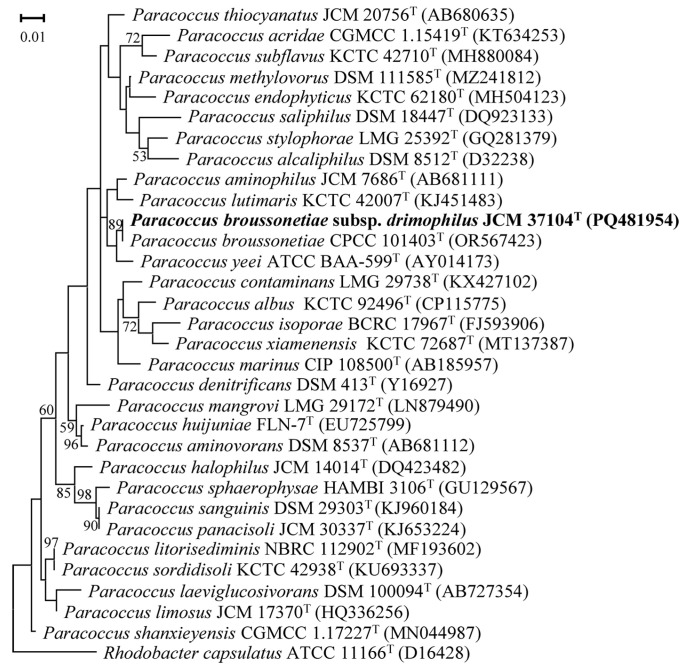
Maximum likelihood (ML) phylogenetic tree based on 16S rRNA gene evolutionary distances among strain NGMCC 1.201697^T^ and members of the genus *Paracoccus*. Bootstrap values (expressed as percentages of 1000 replications) above 50% are shown at branch points. Bar 0.01 substitutions per nucleotide position. *Rhodobacter capsulatus* ATCC 11166^T^ (D16428) was used as an outgroup.

Strain NGMCC 1.201697^T^ features a 16S rRNA gene sequence of 1414 bp, which has been deposited in GenBank (accession number: PQ481954). Comparative analyses using the NCBI and EzBio-Cloud database showed that this strain shares the highest sequence similarity with *P. broussonetiae* CPCC 101403^T^ (99.86%), followed by *P. yeei* CCUG 46822^T^ (97.11%) *P. lutimaris* HDM-25^T^ (97.04%), *P. laeviglucosivorans* NBRC 112902^T^ (96.93%), *P. limosus* NB88^T^ (96.81%), *P. mangrovi* gyp-1^T^ (96.81%), while showing less than 96.66% sequence similarity with the type-strains of other *Paracoccus* species. In the maximum likelihood (ML) phylogenetic tree based on the 16S rRNA gene sequences, strain NGMCC 1.201697^T^ clusters with *P. broussonetiae* CPCC 101403^T^ in a distinct clade supported by an 89% bootstrap value, which underscores their close evolutionary relationship. This clade further groups with *P. yeei* ATCC BAA-599^T^, forming a subset nested within a larger clade that includes *P. lutimaris* KCTC 42007^T^ and *P. aminophilus* JCM 7686^T^. These relationships were consistently observed in both the neighbor-joining (NJ) and maximum parsimony (MP) phylogenetic analyses. Collectively, the phylogenetic topology suggests that strain NGMCC 1.201697^T^ and *P. broussonetiae* CPCC 101403^T^ may represent distinct variants of the same species or strains of a single subspecies (Figure 2).

### 3.3. Genomic Features and Phylogenomic Analysis

**Figure 3:**
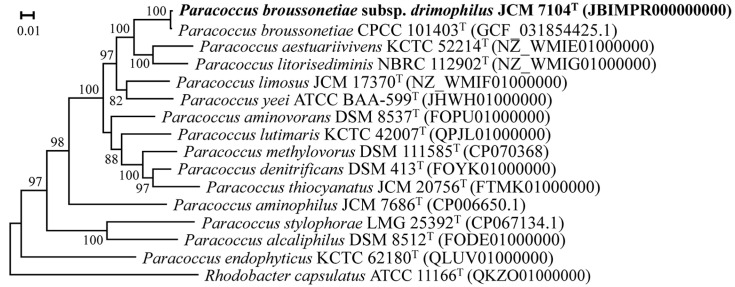
Phylogenomic tree showing the position of strain NGMCC 1.201697^T^ based on bac120 marker set by maximum likelihood inference. Bootstrap values above 50% are shown at the nodes. Bar, 0.01 substitutions per nucleotide position. *Rhodobacter capsulatus* ATCC 11166^T^ (QKZO01000000) was used as an outgroup.

To further elucidate the phylogenomic position of strain NGMCC 1.201697^T^, a maximum likelihood phylogenomic tree was constructed using 120 ubiquitous single-copy marker genes (bac120 marker set) (Figure 3). Core genome analysis positioned strain NGMCC 1.201697^T^ within the *P. broussonetiae* CPCC 101403^T^ clade, forming a highly supported cluster. Moreover, strain NGMCC 1.201697^T^ grouped closely with other type-strains, including *P. aestuariivivens* KCTC 52214^T^ and *P. litorisediminis* NBRC 112902^T^. These results strongly suggest that strain NGMCC 1.201697^T^ and *P. broussonetiae* CPCC 101403^T^ belong to the same species. In contrast, a phylogenetic tree based on the 16S rRNA gene depicted a slightly different topology, with strain NGMCC 1.201697^T^ grouping with both *P. broussonetiae* CPCC 101403^T^ and *P. yeei* ATCC BAA-599^T^. This topological incongruence highlights the limitations of 16S rRNA sequences in capturing high-resolution genomic relationships compared to core genome-based approaches. The dDDH values between strain NGMCC 1.201697^T^ and the type-strains listed in Table 2 were 87.90% for *P. broussonetiae* CPCC 101403^T^*,* 23.70% for *P. litorisediminis* NBRC 112902^T^, 22.70% for *P. yeei* CCUG 46822^T^, 22.60% for *P. denitrificans* ATCC 19367^T^, and 22.30% for *P. aestuariivivens* NBRC 111993^T^. The ANI value was 98.57, 80.96, 80.22, 79.63, and 79.48%, respectively. Although the 98.57% ANI slightly exceeds the conventional subspecies threshold (98.0%) [47], the dDDH result (87.90%), in conjunction with observed physiological and biochemical differentiations, supports the designation of strain NGMCC 1.201697^T^ as a subspecies of *P. broussonetiae* CPCC 101403^T^.

**Figure 4:**
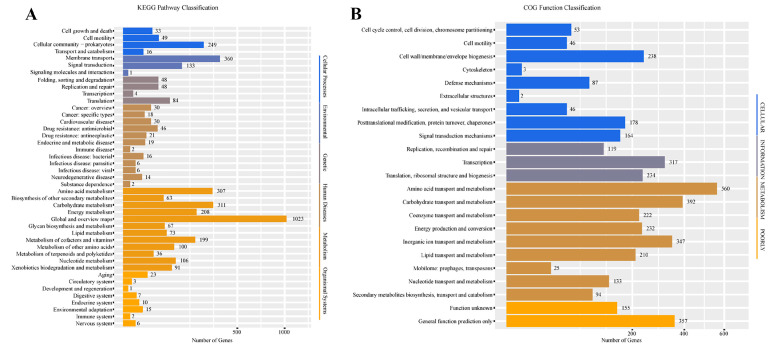
Analysis of the KEGG function classification (**A**), and analysis of the COG function classification (**B**) based on the genomes of stain NGMCC 1.201697^T^.

The draft genome of strain NGMCC 1.201697^T^ comprises 42 contigs, totaling 4,606,090 bp with an average G + C content of 64.49%, consistent with the reported range (63–71 mol%) for the *Paracoccus* species [48,49]. The genome assembly shows an N50 of 236,418 bp, a coverage of 271×, and contains 52 tRNAs, together with one each of 5S rRNA, 16S rRNA, 23S rRNA, and 39 sRNAs. This genome sequence has been deposited in GenBank (accession number: JBIMPR000000000). Functional annotation, based on the KEGG and COG databases (Figure 4), revealed that a considerable number of genes are allocated to several key metabolic pathways. KEGG analysis assigned 1023 genes to global and overview maps, 360 genes to membrane transport, 311 genes to carbohydrate metabolism, 307 genes to amino acid metabolism, and 249 genes to cellular community prokaryotes. Meanwhile, COG classification indicated that the genome is rich in genes related to amino acid transport and metabolism (560 genes), followed by those involved in carbohydrate transport and metabolism (392 genes), genes associated with general function (357 genes), and inorganic ion transport and metabolism (347 genes). Additionally, genes involved in transcription (317 genes); translation, ribosomal structure and biogenesis (234 genes); and energy production and conversion (232 genes) were also notably represented. These genomic features collectively highlight the metabolic diversity and ecological adaptability of strain NGMCC 1.201697^T^, as evident from the enrichment of genes involved in nutrient transport, energy production, and broader metabolic pathways. Additionally, numerous genes involved in transcription (317 genes), translation, ribosomal structure and biogenesis (234 genes), and energy production and conversion (232 genes) further highlight the organism’s metabolic capacity. Collectively, these genomic features display the metabolic diversity and ecological adaptability of strain NGMCC 1.201697^T^, which aligns well with the general adaptability observed in members of the *Paracoccus* genus.

### 3.4. Functional Genomics Analysis

To elucidate the molecular mechanisms underlying salt tolerance, comprehensive genomic analysis of strain NGMCC 1.201697^T^ reveals a sophisticated framework for hypersaline adaptation in Table S1. The analysis primarily focuses on three key physiological mechanisms: osmoregulation processes, ion homeostasis maintenance, and stress-responsive regulatory pathways. The strain NGMCC1.201697^T^ carries a two-component regulatory cascade (*kdpDE-kdpABC*) that plays a central role in its stress response. Here, the sensor kinase (*KdpD*) and response regulator (*KdpE*) activate the *kdpABC* operon, driving high-affinity K+ uptake. This mechanism counters Na^+^ toxicity and is imperative for maintaining cellular turgor and enzymatic stability in environments with fluctuating ionic conditions. Ion homeostasis strategy of the strain NGMCC1.201697^T^ is dual-pronged [50]. Sodium detoxification is achieved by the Na+/H+ antiporter (*nhaA, nhaB*) and a multi-subunit transporter (*mrp*) that exploits the proton gradient (ΔpH) to expel intracellular Na+, thereby contributing to cytoplasmic pH balance. Additionally, the *NhaH*-mediated ion exchange system serves as a primary defence against high salinity. To ensure intracellular K^+^ retention, the strain utilizes both a high-affinity ATP-dependent transporter (*kdpABC*) and a constitutively active symporter (*trkAH*). This synergistic interplay between Na+ expulsion and K+ retention ensures ionic equilibrium, a hallmark of halotolerant adaptation. Complementary to ion regulation, strain NGMCC1.201697^T^ encodes pathways for biosynthesis and uptake of compatible solutes [51,52,53,54]. Genomic evidence identifies glycine betaine as a key osmoprotectant, synthesized by the *betABI-gbsAB* operon and imported through ABC transporters *OpuA-OpuD*. Similarly, the pathways for trehalose biosynthesis (via *otsAB*) and proline synthesis (mediated by *proABC*) were identified, both of which stabilize macromolecular structures under desiccation or ionic stress. Trehalose’s ability to form amorphous glass matrices at high concentrations suggests a dual role in membrane protection and desiccation resistance, while elevated proline levels counteract the dehydration effect caused by extracellular high salt levels through osmoregulation. The integration of these mechanisms not only buffers cytoplasmic osmolarity, but also minimizes oxidative and structural damage, ensuring metabolic continuity under sustained salinity. The prominence of these pathways in strain NGMCC1.201697^T^, compared to genomic annotations of related species, underscores evolutionary specialization toward high-salinity niches. Future studies correlating transcriptional dynamics of these genes under salt stress could further elucidate their regulatory hierarchy and ecological relevance.

Endophytic microbiota represent a critical micro-ecosystem that is pivotal for crop growth and environmental stress response. In the case of strain NGMCC 1.201697^T^, its ability to grow under low-temperature conditions is underpinned by a suite of genomic features (Table S2) that collectively contribute to efficient cold adaptation. On ice-binding proteins (IBPs), the presence of the *ibpA* gene, which encodes the antifreeze glycoproteins, is key to the strain’s cold-resilience. These ice-binding proteins interfere with ice crystal formation by attaching to ice surfaces via their β-helical domains, thereby inhibiting crystal growth and lowering the freezing point of intracellular water. Beyond this, some IBPs (such as bacterial MpAFP) also contribute to membrane stabilization by protecting the lipid bilayer against phase transitions at low temperatures. Direct studies on IBPs in *Paracoccus* species are limited, but they have been reported in other organisms. For example, the MpAFP of *Marinomonas primoryensis* binds to ice surfaces through repetitive patterns in its β-helical domain [55]. Low-temperature response regulators have also been identified in the strains: cold shock proteins (*cspA*), cold adaptation proteins (*cap*) [56], and DNA-binding proteins (*hns*). *cspA*s encode cold shock proteins that contribute to the stabilization of mRNA under low-temperature conditions and facilitate the rapid transcriptional response required for bacterial adaptation to cold environments [57]. Furthermore, cold adaptation proteins (*cap* genes) were also characterized, which are crucial for maintaining membrane fluidity and integrity at lower temperatures [58]. The interplay between CSPs and CAPs not only supports bacterial viability in cold environments, but can also enhance plant growth and resilience when associated with host plants. *hns* encode DNA-binding proteins, and H-NS (histone-like nucleoid structuring protein) plays a critical role as a global regulatory factor. Under low-temperature response effects, the binding affinity of the H-NS protein to DNA has changed. At low temperatures (<20 °C), a stable nucleoid complex is formed; at high temperatures (>37 °C), domain dissociation occurs, releasing the bound DNA. IBPs protect cellular structures, such as membrane integrity, by inhibiting ice crystal growth, while CSPs/CAPs maintain metabolic activity. Together, these genomic adaptations not only enable strain NGMCC 1.201697^T^ to withstand and thrive under cold conditions, but also likely contribute to its beneficial interactions as an endophyte, enhancing plant growth and stress responses.

The environmental burden of persistent plastics has intensified global efforts to identify biodegradable alternatives. Among these, polyhydroxyalkanoates (PHAs), microbially synthesized linear polyesters, stand out as environmentally sound substitutes due to their conventional thermoplastic properties and enzymatic degradability in diverse ecosystems. Bacteria represent a potential vehicle for PHA biosynthesis. A complete PHA biosynthesis operon (*phaA-G*), alongside a depolymerase gene (*phaZ*), was identified from strain NGMCC 1.201697^T^ (Table S1), suggesting competence for PHA esters synthesis. The type species of the genus has been well-explored for PHA synthesis [59], with some strains found to predominantly accumulate homopolymers of polyhydroxybutyrate (PHB) [60] or polyhydroxyvalerate (PHV) [61], as well as copolymers of P(HB-co-HV) [62]. Strain NGMCC 1.201697^T^ could be a potential PHA-producing strain. Further experiments will be performed to elucidate the conditions for the PHA production by NGMCC 1.201697^T^.

### 3.5. Chemotaxonomic Characteristics

**Figure 5:**
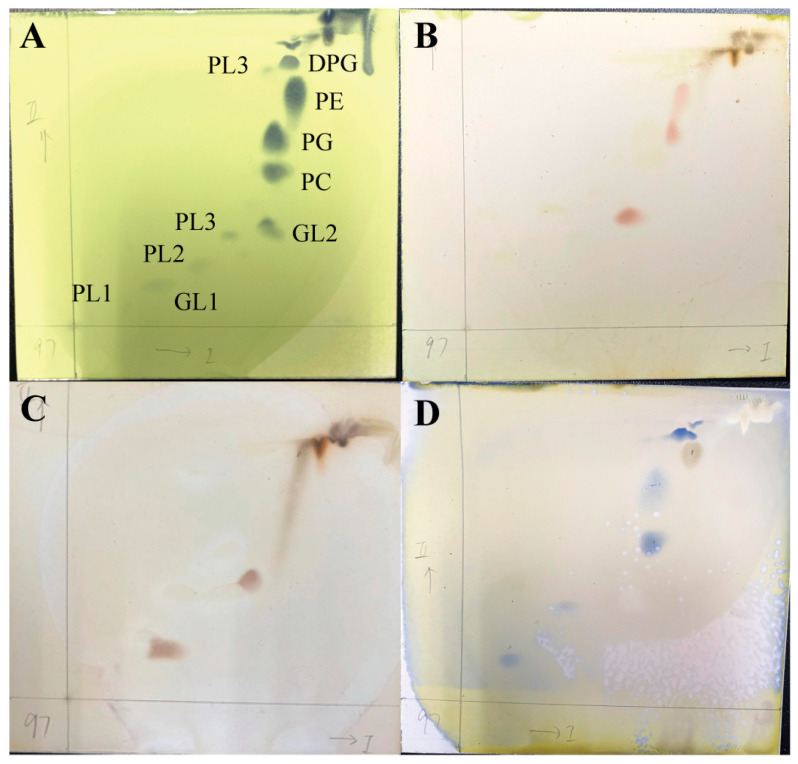
Polar lipids profile of strain NGMCC 1.201697^T^. The plates in Figure 5 (**A**–**D**) are sprayed with molybdophosphoric acid, ninhydrin, α-naphthol and molybdenum blue staining agent to show all polar lipids present, respectively. DPG, diphosphatidylglycerol; PE, phosphatidylethanolamine; PC, phosphatidylcholine; PG, phosphatidylglycerol; GL1–2, two unidentified glycolipids; PL1–4, unidentified phospholipid.

The fatty acid profile of strain NGMCC 1.201697^T^ is dominated by summed feature 8 (C_18:1_ *ω7c*, 69.42%), with C_16:0_ (15.94%) and C_18:0_ (6.75%) as minor constituents (Table 3). In contrast, the closest related strain, *P. broussonetiae* CPCC 101403^T^, exhibited a lower proportion of summed feature 8 (C_18:1_ *ω7c*, 63.3%) alongside a higher C_16:0_ (20.1%). The degree of unsaturation in fatty acids is related to the fluidity of cell membranes. Unsaturated fatty acids help maintain membrane fluidity at low temperatures, preventing membrane stiffening, which is crucial for cold tolerance. Summed feature 8 is a type of monounsaturated fatty acid, and increasing its proportion may enhance membrane fluidity. The 6.1% increase in summed feature 8, paired with reduction in C_16:0_, likely indicates adjustments of membrane lipid composition to environmental stresses such as osmotic pressure and temperature consistent with the observed superior growth of strain NGMCC 1.201697^T^ at 4 °C. Significant differences in lipid unsaturation are observed, which may underpin their distinct cold adaptation capabilities. Although the proportions of fatty acids in strain NGMCC 1.201697^T^ differed from those in the four closest related type-strains, the major fatty acid profiles remained consistent, indicating a shared feature within the genus. In terms of respiratory quinones, both strains predominantly contain ubiquinone-10 (Q-10), which is recognized as the predominant respiratory quinone among all *Paracoccus* species. Minor traces of Q-9 and Q-11 were occasionally detected in some species, whereas Q-8 has been reported as the major ubiquinone *Paracoccus yeei* [63]. Regarding polar lipids, strain NGMCC 1.201697^T^ exhibited the polar lipid profile (Figure 5) consisting of diphosphatidylglycerol (DPG), phosphatidylglycerol (PG), phosphatidylcholine (PC), phosphatidylethanolamine (PE), two unidentified glycolipids (GLs) and four unidentified phospholipids (PLs). This is notably similar to the profile of strain *P. broussonetiae* CPCC 101403^T^, which contains PG, DPG, PC, and uncharacterized GL. Four unidentified phospholipid (PLs) and PE were present in strain NGMCC 1.201697^T^, and AL was absent in strain *P. broussonetiae* CPCC 101403^T^ (Table S2). These differences may reflect subspecies-level functional divergence.

## 4. Conclusions

In conclusion, on the basis of phenotypic, chemotaxonomic, genotypic, and phylogenetic studies, strain NGMCC 1.201697^T^ can be considered as a novel subspecies in the genus *Paracoccus*, which we propose to name *Paracoccus broussonetiae* subsp. Drimophilus, subsp. nov.

### Description of Paracoccus broussonetiae subsp. drimophilus subsp. nov.

*Paracoccus broussonetiae* subsp. *drimophilus* (‘*drimophilus*’ is. L. gen. n. drimophilous, referring to the salt tolerance of type-strain NGMCC 1.200843^T^).

Cells are Gram-negative, aerobic, non-motile, non-spore-forming, short rod-shaped, and typically arranged in pairs, with a major axis of 0.60–0.85 μm and minor axis of 0.55–0.60 μm. When cultured on LB agar for 2 days, colonies are 1.0–2.0 mm in diameter, yellowish-white, circular, glistening, and convex with intact edges. The strain grows optimally at 30 °C, with a temperature range spanning from 4 °C to 37 °C. The optimal pH is 7.0, with growth at pH 4.0–10.0. Growth is supported in 0–8.0 % (*w*/*v*) NaCl, with the optimum at 1.0 %. In assays with the API ZYM system, alkaline phosphatase, esterase (C4), lipid esterase (C8), leucine arylamidase, valine arylamidase, cystine arylamidase, acid phosphatases, catalase, and naphthol-AS-BI-phosphohydrolase are present, but lipoidase (C14), trypsin, chymotrypsin, α-galactosidase, β-galactosidase, β-glucuronidase, α-glucosidase, β-glucosidase, N-Acety-β-glucosaminidase, α-mannosidase, and α-fucosidase are absent. In the API 20NE test, cells are positive for nitrate assimilation, urea, esculin hydrolyse, D-glucose assimilation, L-arabinose, D-mannose, D-mannitol, N-acetyl-glucosamine, D-maltose (weak), potassium gluconate (weak), adipate (weak), L-malate, phenylacetate, oxidase. The major isoprenoid quinone is Q-10. The predominant cellular fatty acid is summed feature8 (C_18:1_ *ω7c*) and C_16:0_. The major polar lipids are DPG, PG, PC, PE, GLs, and PL. The DNA G + C content is 64.49 mol%. The type-strain, NGMCC 1.201697^T^ (=CGMCC 1.61958^T^ =JCM37104^T^), was isolated from maize roots in Hunan Province, China.

## Figures and Tables

**Table 1:** Comparative analysis of physiological and biochemical features between strain NGMCC 1.201697^T^ and its closest *Paracoccus* relatives. Strains: 1, NGMCC 1.201697^T^; 2, *Paracoccus broussonetiae* CPCC 101403^T^; 3, *Paracoccus litorisediminis* NBRC 112902^T^; 4, *Paracoccus yeei* ATCC BAA-599^T^; 5, *Paracoccus denitrificans* ATCC 19367^T^. Data presented were derived from this investigation unless otherwise cited. Symbols: +, positive; −, negative; w, weakly positive; v, variable; ND, no data.

Characteristics	1	2	3	4	5
Color	pale yellow	semitranslucent	yellowish white	white gray	pale yellow
NaCl range (%)	0–8.0	0–5.0	0–5.0	4.0–6.0	0–7.0
Optimal NaCl (%)	0–2.0	0–1.0	2.0	4.0	ND
Temperature range	4–37	10–37	10–40	21–37	20–40
Optimal temperature	30	30	30	30	30
pH range	4–10	7.0–9.0	5.5–9.5	5.0–8.5	5.5–9.0
Optimal pH	7.0	7.0	7.0–8.0	7.0	ND
Esterase (C4)	+	−	+	+	+
Trypsin	−	+	−	−	−
α-glucosidase	v	−	+	+	−
N-Acety-β-glucosaminidase	v	−	+	−	−
NO3	+	−	+	+	+
D-maltose	w	−	−	−	+
citrate	−	−	+	+	+
β-Glucosidase	+	−	+	−	−
D-Mannitol	+	−	ND	+	+
D-sorbitol	+	−	ND	+	+
saccharose/sucrose	+	−	−	−	+
D-Trehalose	+	−	−	−	+
α-galactosidase	+	−	+	ND	−
L-Histidine	−	+	ND	−	+
L-lactate	−	+	ND	w	+

**Table 2:** The average nucleotide identity (ANI) and digital DNA–DNA hybridization (dDDH) values between the strain NGMCC 1.201697^T^ and closest relatives *Paracoccus*. Genome of strain NGMCC 1.201697^T^ Genbank accession number is JBIMPR000000000.

Query Genome	Reference Genome	ANI (%)	dDDH (%)
NGMCC 1.201697	*Paracoccus yeei* CCUG 46822^T^ (CP024422.1)	80.22	22.70
NGMCC 1.201697	*Paracoccus denitrificans* ATCC 19367 ^T^ (CP035092.1)	79.63	22.60
NGMCC 1.201697	*Paracoccus lutimaris* CECT 8525^T^ (QPJL01000001.1)	79.19	21.90
NGMCC 1.201697	*Paracoccus aestuariivivens* NBRC 111993^T^ (NZ_WMIE01000001.1)	79.48	22.30
NGMCC 1.201697	*Paracoccus litorisediminis* NBRC 112902^T^ (NZ_WMIG01000001.1)	80.96	23.70
NGMCC 1.201697	*Paracoccus thiocyanatus* ATCC 700171^T^ (FTMK01000001.1)	79.53	22.20
NGMCC 1.201697	*Paracoccus broussonetiae* CPCC 101403 ^T^ (JAVRQI000000000)	98.57	87.90

**Table 3:** Cellular fatty acid compositions of strains NGMCC 1.201697^T^ and three closest related type *Paracoccus* species. Strains: 1, NGMCC 1.201697^T^; 2, *Paracoccus broussonetiae* CPCC 101403^T^; 3, *Paracoccus litorisediminis* NBRC 112902^T^; 4, *Paracoccus yeei* CCUG 46822^T^; 5, *Paracoccus denitrificans* ATCC 19367^T^. Values ≥ 1% are shown.

Fatty Acid	1	2	3	4	5
**Saturated Straight-Chain**					
C_16:0_	15.94	20.1	9.2	13	9.4
C_17:0_	―	―	3.3	1	0.7
C_18:0_	6.75	1.6	3.2	5	1.9
C_18:2_	―	―	―	1	―
iso-C_16:0_	―	1.0	―	―	―
**Unsaturated Straight-cChain**					
C_18:1_ *ω7c*	69.42	63.3	79.7	71	74
C_17:1_ *ω8c*	―	1.0	―	―	―
C_18:1_ *ω9c*	―	―	―	1	―
C_12:1_ *ω7c*	―	―	―	3	―
Cyclo C_19:0_ *ω8c*	1.39	3.2	―	―	7.8
**Hydroxy**					
C_10:0_ 3-OH	2.10	―	2.9	3	2.8
C_14:0_ 3-OH	―	―	―	1	―
**Summed Features**					
Summed Feature 2	1.46	1.8	2.2	―	2.6
Summed Feature 3	―	1.4	―	―	―
Summed Feature 4	―	3.0	―	―	―
Summed Feature 8	69.42	63.3	79.7	71	74

Summed features represent groups of two or three fatty acids that cannot be separated by the Microbial Identification System. Summed feature 2 consisted of C_12:0_ aldehyde and/or an unknown fatty acid ECL 10.9525. Summed feature 8 contained C_18:1_ *ω6c* and/or C_18:1_ *ω7c*. Summed feature 3 comprises C_16:1_ *ω7c* and/or C_16:1_ *ω6c*. Summed feature 4 comprises anteiso-C_17:1_B/iso-C_17:1_ I.

## Data Availability

The GenBank accession number for the 16S rRNA gene sequence of strain NGMCC 1.201697^T^ is PQ481954. This whole genome shotgun project has been deposited in GenBank under the accession JBIMPR000000000.

## Supplementary Materials

The following are available online at https://www.mdpi.com/article/10.3390/life15030354/s1, Table S1: Identified genes in strain NGMCC 1.201697^T^ genome for salt tolerance and stress response. Table S2: Comparative physiological and biochemical features between strain NGMCC 1.201697^T^ and type-strains of the closest related *Paracoccus* species.
