# Supplementary material for: Paracoccus broussonetiae subsp. drimophilus subsp. nov., a Novel Subspecies Salt-Tolerant Endophytic Bacterium from Maize Root in Hunan
Source: Life (Basel). 2025 Feb 24;15(3):354. doi: 10.3390/life15030354 (PMC11944123; doi:10.3390/life15030354)
Supplement: Supplementary file 1 [file life-15-00354-s001.zip › Supplement Table S2.pdf]

Table S2. Comparative physiological and biochemical features between strain NGMCC 1.201697^T and type strains of the closest related *Paracoccus* species. Strains: 1, NGMCC 1.201697^T; 2, *Paracoccus broussonetiaeyeei* CPCC 101403^T; 3, *Paracoccus litorisediminis* NBRC 112902^T; 4, *Paracoccus yeei* ATCC BAA-599^T; 5, *Paracoccus denitrificans* ATCC 19367^T. Data were obtained in this study unless indicated. +, Positive; -, negative; w, weakly positive; v, variable; ND, no data.

Characteristics	1	2	3	4	5
Colour	Pale yellow	semitranslucent	yellowish white	white–gray	pale yellow
NaCl range (%)	0–8.0	0–5.0	0–5.0	4.0–6.0	0–7.0
Optimal NaCl	1.0	0–1.0	2.0	4.0	ND
Optimal temperature	30	30	30	30	30
Temperature range	4–37	10–37	10–40	21–37	20–40
Optimal pH	7.0	7.0	7.0–8.0	7.0	ND
pH range	4–10	7–9	5.5–9.5	5.0–8.5	5.5–9.0
G+C (mol%)	64.49	64.0	64.1	67.4	66.5
polar lipid	DPG, PG, PC, PE, 2 GLs, 4 PLs	DPG, PG, PC, 3 GL, 1 AL	PC, PG, DPG, 1 AL, 1 GL	ND	PG, PE, PC, CL, OL
**API ZYM results**					
Alkaline phosphatase	+	ND	+	+	+
Esterase(C4)	+	–	+	+	+
Lipid esterase(C8)	+	+	+	+	–
Lipoidase(C14)	–	–	–	–	–
Leucine arylamidase	+	ND	+	+	+
Valine arylamidase	+	ND	+	–	+
Cystine arylamidase	+	ND	+	–	w
Trypsin	–	+	–	–	–
Chymotrypsin	–	–	–	–	–
Acid phosphatases	+	ND	+	+	+
Naphthol-AS-BI-phosphohydrolase	+	+	+	+	+
α-galactosidase	–	–	–	–	–
β-galactosidase	–	–	–	–	–
β-glucuronidase	–	–	–	–	–
α-glucosidase	–	–	+	+	–
β-glucosidase	–	–	–	–	–
N-Acety-β-glucosaminidase	–	–	+	–	–
α-mannosidase	–	–	–	–	–
α-fucosidase	–	–	–	–	–
catalase	+	+	+	+	+
**API 20NE**					
NO3(nitrate assimilation)	+	–	+	+	+
TRP(L-tryptophane)	–	ND	ND	–	–
GLU(fermentation D-glucose)	–	ND	+	+	+
ADH(Arginine DiHydrolase)	–	–	ND	–	w

URE(urease)	+	ND	ND	+	+
ESC(esculin hydrolyse)	+	ND	–	–	–
GEL(gelatin)	–	–	–	–	–
PNPG(Para-nitrophenyl-ßD-galactopyranosidase)	–	ND	ND	ND	ND
GLU(assimilation D-glucose)	+	+	+	+	+
ARA(L-arabinose)	+	ND	+	+	–
MNE(D-mannose)	+	+	+	+	+
MAN(D-mannitol)	+	–	ND	+	+
NAG(N-acetyl-D-glucosamine)	+	+	ND	–	+
MAL(D-maltose)	w	–	–	–	+
GNT(potassium gluconate)	w	ND	ND	–	+
CAP(caprate)	–	ND	ND	ND	ND
ADI(adipate)	w	ND	ND	ND	w
MLT(L-malate)	+	+	+	+	+
CIT(citrate)	–	–	+	+	+
PAC(phenylacetate)	+	ND	ND	–	+
OX(oxidase test)	+	+	+	+	+
**VITEK 2 GN**					
APPA(Ala-Phe-Pro Arylamidase)	–	ND	ND	ND	ND
ADO(adonitol)	+	ND	ND	+	ND
PyrA(L-Pyrrolydonyl-arylamidase)	+	ND	ND	ND	ND
lARL(L-Arabitol)	+	+	ND	+	ND
dCEL(D-cellobiose)	+	–	ND	+	–
BGAL(β-galactosidase)	+	–	ND	ND	–
H2S(H2S production)	–	–	ND	ND	ND
BNAG(β-N-acetyl-glucosaminidase)	–	–	+	–	–
AGLTp(Glutamyl Arylamidase pNA)	–	ND	ND	ND	ND
dGLU(D-Glucose)	+	+	+	+	+
GGT(γ-glutamyl-transferase)	–	ND	ND	ND	ND
OFF(Fermentation/glucose)	+	ND	+	+	+
BGLU(β-Glucosidase)	+	–	+	–	–
dMAL(D-maltose)	+	–	–	–	+
dMAN(D-Mannitol)	+	–	ND	+	+
dMNE(D-mannose)	+	+	+	+	+
BXYL(β-Xylosidase)	+	ND	+	ND	–
BAlap(β-Alanine arylamidase pNA)	–	ND	ND	ND	ND
ProA(L-Proline arylamidase)	+	ND	ND	–	+
LIP(Lipase)	–	–	–	–	–
PLE(Palatinose)	+	ND	ND	ND	ND
TyrA(Tyrosine Arylamidase)	+	ND	ND	ND	+

URE(Urease)	+	ND	ND	+	+
dSOR(D-sorbitol)	+	–	ND	+	+
SAC(saccharose/ sucrose)	+	–	–	–	+
dTAG(D-Tagatose)	+	ND	ND	+	ND
dTRE(D-Trehalose)	+	–	–	–	+
CIT(citrate(sodium))	+	ND	+	+	+
MNT(Malonate)	+	ND	ND	ND	ND
5KG(5-Keto-d-Gluconate)	–	ND	ND	–	–
lLATk(L-Lactate alkalinisation)	+	ND	ND	ND	+
AGLU(α-glucosidase)	v	–	+	+	–
SUCT(Succinate alkalinisation)	+	ND	+	ND	+
NAGA(β-N-Acetyl-Galactosaminidase)	+	+	ND	–	ND
AGAL(α-galactosidase)	+	–	+	ND	–
PHOS(phosphatase)	+	ND	ND	ND	ND
GlyA(Glycine Arylamidase)	+	ND	ND	ND	+
ODC(Ornithine Decarboxylase)	+	ND	ND	ND	ND
LDC(Lysine Decarboxylase)	–	ND	ND	ND	w
lHISa(L-Histidine assimilation)	–	+	ND	–	+
CMT(Courmarate)	+	ND	ND	ND	ND
BGUR(β- glucuronidase)	–	–	–	–	–
O129R(O/129 resistance)	+	ND	ND	ND	ND
GGAA(Glu-Gly-Arg-Arylamidase)	–	ND	ND	ND	ND
lMLTa(L-Malate assimilation)	–	ND	+	+	+
ELLM(ELLMAN)	+	ND	ND	ND	ND
lLATa(L-lactate assimilation)	–	+	ND	w	+
